# Broader Considerations of Higher Doses of Donepezil in the Treatment of Mild, Moderate, and Severe Alzheimer's Disease

**DOI:** 10.1155/2012/707468

**Published:** 2011-12-01

**Authors:** Camryn Berk, Marwan Sabbagh

**Affiliations:** The Cleo Roberts Center for Clinical Research, Banner Sun Health Research Institute, 10515 W. Santa Fe Dr, Sun City, AZ 85351, USA

## Abstract

Donepezil, a highly selective acetylcholinesterase inhibitor (AChEI), is approved as a symptomatic treatment mild, moderate, and severe Alzheimer's disease (AD). Donepezil exerts its treatment effect through multiple mechanisms of action including nicotinic receptor stimulation, mitigation of excitotoxicity, and influencing APP processing. The use of donepezil at higher doses is justified given the worsening cholinergic deficit as the disease advances. Donepezil has been investigated in several clinical trials of subjects with moderate-to-severe AD. While the side effects are class specific (cholinergically driven), demonstrable benefit has been shown at the 10 mg dose and the 23 mg doses. Here, we review the clinical justification, efficacy, safety, and tolerability of use of donepezil in the treatment of moderate-to-severe AD.

## 1. Introduction

Alzheimer's disease is an age-related progressive neurological disorder that ultimately results in cognitive and behavioral dysfunction and functional loss. These later stages of disease are impacting an increasingly large number of people each year. At present, the treatment of Alzheimer's disease (AD) is limited to symptomatic therapy. As symptoms worsen and the effects of late-stage Alzheimer's disease become more severe, there is an urgent need for new and novel treatment options. Of the 5.3 million people estimated to be afflicted with AD in the United States, more than half are suspected to have moderate-or-severe disease. Because these stages of AD can last for several years, the difficulty presented to both patients and caregivers increases dramatically in advanced stages [1].

 Donepezil, a highly selective acetylcholinesterase inhibitor (AChEI), is one of only two treatments approved in the United States for use beyond the mild-to-moderate stage. While it can slow progression modestly and treat symptoms, it cannot halt progression of the actual disease or ultimately stop the decline in cognitive and functional abilities. Agents such as donepezil create notable symptomatic improvements with modest but debatable impact on disease progression from mild to moderate is concerned, decreasing speed of progression from the earliest signs of cognitive impairment to severe dementia and finally death [2, 3].

An important component of Alzheimer's disease management is the group of caregivers who support Alzheimer's patients. As Alzheimer's disease progresses, the demands on the caregiver increase; often this takes a toll on the care provider in the form of health problems of their own, stress, anxiety, depression, fatigue, and other physical and emotional problems [4]. Donepezil is a valuable treatment option not just for its capacity as a selective inhibitor of acetylcholinesterase for the patients but also for its capacity to ease the burden of the caregiver by prolonging the AD patient's ability to perform self-care tasks and delaying the progression of symptoms that would impact a caregiver [5].

## 2. Overview

Donepezil has a mode of action based on inhibiting acetylcholinesterase selectively. Cholinergic function is improved when there are greater concentrations of acetylcholine in the brain, and the depletion of acetylcholine that is common with AD patients is positively affected with a donepezil regimen. Additionally, cholinesterase inhibitors hypothetically could operate within the scope of the “amyloid hypothesis” of AD pathogenesis. Donepezil in particular has been shown to attenuate neurotoxicity of amyloid beta peptide A*β* as well as influencing APP processing [6]. In accordance with the cascade concept, therapies that directly target the accumulation, aggregation, and deposition of amyloid beta peptide A*β*_42_ and effectively reduce the inciting event may be more effective than distally targeted treatments, such as those that target neurotransmitter deficits.

In addition to attenuating A*β* neurotoxicity, donepezil also has an effect on cholinergic deafferentation. Cell bodies in the nucleus basalis in the brain are the origin of cholinergic tracts, the axons of which are prolific in the cerebral cortex and in the pedunculopontine nucleus with expansion into the thalamus. Loss of cholinergic neurons in the nucleus basalis are a hallmark of the earliest stages of AD. The functional deafferentation of the cholinergic neurons is thought to induce A*β* production in the cerebral cortex, which is what begins the cascade to neurodegeneration. Donepezil disrupts the mechanism by which A*β* generation occurs following cholinergic deafferentation by interfering with the process that leads to the exposing of the toxic N-terminal region of APP during the preferential processing of the amyloid precursor protein. One way donepezil interupts this process is by cholinergic input to cortical neurons [7].

Donepezil also impacts nicotinic receptors in the cortex through upregulation. At clinically relevant concentrations, donepezil decreases the reduction of nicotinic acetylcholine receptor expression in the cerebral cortex of AD patients and prevents some of the reduction in nicotinic binding that correlates to disease severity. This process reduces glutamate neurotoxicity and also has been shown to inhibit excitotoxic injury, which is significant regarding AD pathogenesis and progression [8].

Other mechanisms of action indicate donepezil could provide a treatment option for affecting the cellular and molecular processes of neurodegeneration rather than symptomatic treatment. Donepezil has been found to influence acetylcholinesterase isoform expression by inhibiting the expression of the AChE-S form and causing an increase in the expression of the AChE-R form through reduction of cholinesterase, generating neuroprotective effects [9]. Donepezil has also been shown to upregulate nicotinic receptors in the cerebral cortex, increasing nicotinic acetylcholine receptor expression and nicotinic binding [10]. Furthermore, under conditions of upregulations donepezil has been proven to maintain neuroprotective actions are lower drug concentrations and reduce glutamate toxicity [8]. Donepezil has been shown to mitigate the effects of oxidative stress in a streptozotocin-induced model of dementia in mice, indicating use against free radicals that may be implicated in AD [11]. Donepezil treatment has also resulted in improved cerebral blood flow in patients with mild cognitive impairment, with no reduction after six months compared to statistically significant reduction in the placebo cohort [12, 13]. Potential mechanisms of action also include enhancing neuroplastic activity through cholinergic modulation. In rodent models of basocortical degeneration, donepezil induced cortical sprouting, mitigating reduction in cholinergic neurotransmission [14].

## 3. Efficacy

Donepezil has been found to be one of the most effective symptomatic treatments for AD available on the market with additional potential as a disease-modifying therapy. Many but not all studies have shown that both when compared to a placebo, donepezil has proven to have significant efficacy in reducing the severity of neuropsychiatric symptoms in patients with mild-to-moderate AD and patients in more severe stages of the disease [15]. Additionally, donepezil has been found to be effective in symptomatic treatment in a variety of dosing levels giving it variability as a treatment option. In an open-label, multicenter, Phase 3 extension study of the safety and efficacy of donepezil in patients with Alzheimer's disease [15], the primary efficacy measures were the Alzheimer's Disease Assessment Scale-cognitive subscale (ADAS-Cog) and the CDR-Sum of the Boxes (CDR-SB). These measures were evaluated in a 152-week long extension study on patients aged 50 and older that participated in at least one of the previous phases of donepezil trials, with a total patient number (n) of 763. Among this population, the most common reason for study discontinuation (46% of discontinued patients) was departure due to the commercialization of donepezil after the successful completion of nonextension trials, allowing for prescriptive use of the drug [15], but of those that completed the study declined from baseline significantly less than the placebo arm in the same period with regards to ADAS-Cog scores. After a three-week placebo washout, the mean change for patients using CDR-SB scores actually improved above baseline for the experimental arm and declined in the placebo arm.

In comparison studies with rivastigmine and galantamine, specifically a 52-week trial [16] and a 12-week trial [17] showed that regarding cognitive function, there is no statistically significant difference between patients treated with donepezil versus those treated with galantamine, nor is there a difference regarding behavior when comparing 10 mg/day of donepezil to 24 mg/day of galantamine. Similarly, rivastigmine and donepezil have been found to have comparable effects on cognition and behavior [18, 19] and similar gastrointestinal side effects profiles [20].

## 4. Safety and Tolerability

In patients with moderate-to-severe Alzheimer's disease, donepezil has been found to be safe and well tolerated at doses up to 23 mg, with an increase in adverse events over lower dosages, most of which dissipate after the first month of treatment [1, 21]. In a randomized, double-blind study, 1371 patients (mean age: 73.8 years, 62.8% female, 73.5% white) from 219 different sites in Asia, Europe, Australia, North America, South America, and South Africa were analyzed for comparative effects of 10 mg/day donepezil and 23 mg/day donepezil. All patients had an MMSE of less than 20 and assessed for changes in cognition and global functioning as assessed using least square regressions as well as deviation from baseline between the two cohorts. At the highest doses, the most significant adverse events reported in safety trials were nausea, vomiting, and diarrhea; in the event that these symptoms did not dissipate, dosages were reduced to improve tolerability. Nevertheless, a certain percent of participants did not tolerate donepezil even after the dose was reduced. Donepezil has proven to be safe and well tolerated as well as effective in the treatment of moderate-to-severe AD [21].

Donepezil has been found to be an effective option for maintaining a desirable quality of life and prolonging patient performance of activities of daily living (ADL); under study of 290 (mean age: 73.6 years, range 48–92) patients randomized into a 24-week, double-blind, placebo-controlled study, donepezil demonstrated a vastly slower decline trajectory than placebo in ADLS in patients with moderate-to-severe AD [22]. Using the Disability Assessment for Dementia (DAD), the Instrumental Acitvities of Daily living (IADL) scale, and the Physical Self-Maintenance Scale (PSMS), researchers have determined that moderate-to-severe AD patients taking donepezil were improved in tasks such as the use of household appliances, managing personal mail, moving around in and outside of homes, understanding explanations or new situations, and maintaining leisure activities as compared to moderate-to-severe AD patients who took a placebo [22, 23]. In a six-month study, assessing responder rates of moderate-to-severe AD patients taking donepezil in a long-term care facility, greater proportions of patients defined as responders to donepezil showed significant stabilization or improvement compared with placebo on individual efficacy measures including the severe impairment battery (SIB) (≥0,≥4 or ≥7 points) and Mini-Mental State Examination (≥0 or ≥3 points), and positive trends on the ADCS-ADL-severe (≥3 points) and the neuropsychiatric inventory (NPI) cluster based on mood items. All 3 composite measures of efficacy showed a significantly higher proportion of responders in the donepezil group. The responders had a similar distribution between the 2 subgroups of cognitive and functional disease severity at baseline. The donepezil-treated patients taking psychotropic drugs showed significantly greater improvement on the SIB, less deterioration on the ADCS-ADL, and had higher Clinical Global Impression of Improvement scores and a trend toward lower NPI scores [23].

In addition to assessing patient conditions, caregivers were assessed using the Caregiver Stress Scale (CSS) for the first time in a clinical trial to measure a caregiver's status regarding cognitive status, overload, relational deprivation, job-caregiving conflict, economic strains, role captivity, loss of self, care-giving competence, personal gain, management of distress, and expressive support. Statistical analysis of efficacy was based on change from baseline of MMSE scores. According to the caregiver journal assessments, patients treated with donepezil were more likely to be capable of interacting with caregivers and others, engaging and being interested in conversations with others, and enjoying leisurely activities. Caregivers of donepezil-treated patients remained close to their baseline assessments of stress levels while caregivers for the placebo group reported significant increases in stress over the same period. Treatment of moderate-to-severe AD with donepezil has also been associated with a much more gradual increase in time a caregiver spends caring for a patient with progressing disease compared to patients who received a placebo [24].

As indicated in the aforementioned Doody study, the adverse events experienced when treated with donepezil are transient and mild, often resolving themselves without the need for dose modification but in the rare cases resolution is elusive, lowering the dosage is an effective way to decrease adverse events. 92% of patients in the study experienced at least one treatment-related adverse event, with only 28% (214) of patients experiencing a serious (grade 3 or 4) AE; in the case of 27% (203), the serious nonfatal AEs experienced was determined to be not related to donepezil treatment. Treatment with donepezil also indicated no clinically significant results or changes in clinical laboratory tests, physical examinations, or electrocardiograms. The results of this study indicated that donepezil is a safe and effective agent for long-term treatment of moderate-to-severe disease [15].

## 5. Conclusions

It is broadly recognized that potent disease modifying agents are needed to prevent the progression of Alzheimer's disease. While the “amyloid hypothesis” of AD pathogenesis is still unproven, there is substantial evidence that suggests that inhibiting A*β* plaque formation will be a valuable approach for achieving this goal. But the foundation of AD management will continue to include a cholinesterase inhibitor such as donepezil. A multitude of well-controlled clinical studies have demonstrated the cognitive benefit of this agent. In addition, by interfering with the process that leads to the exposing of the toxic N-terminal region of APP during the preferential processing of the amyloid precursor, donepezil disrupts the mechanism by which A*β* generation occurs following cholinergic deafferentation. As new therapeutics come on line, the next advance in AD patient management will come from understanding how they synergize with donepezil or other cholinesterase inhibitors.

Going forward, the role donepezil plays in disease management should not be limited solely to cholinestrase inhibition at the level of neurotransmitter. Rather, as preclinical evidence amply demonstrates, the drug affects on cellular and molecular processes should be exploited for their depth and breadth of influence at every stage of disease.
